# Blood Flow Restriction: How Does It Work?

**DOI:** 10.3389/fphys.2012.00392

**Published:** 2012-10-04

**Authors:** Jeremy P. Loenneke, Takashi Abe, Jacob M. Wilson, Carlos Ugrinowitsch, Michael G. Bemben

**Affiliations:** ^1^Neuromuscular Research Laboratory, Department of Health and Exercise Science, The University of OklahomaNorman, OK, USA; ^2^Department of Health, Exercise Science, and Recreation Management, University of MississippiOxford, MS, USA; ^3^Department of Health Sciences and Human Performance, University of TampaTampa, FL, USA; ^4^School of Physical Education and Sport, University of São PauloSão Paulo, Brazil

The purpose of this viewpoint is to provide rationale for the cellular investigation of blood flow restriction (BFR) in the absence of exercise, as this may provide a novel insight into the mechanisms exclusive to the restriction stimulus itself. Most of the research on BFR thus far has been completed with BFR in combination with low intensity resistance training. To illustrate, BFR in combination with low intensity exercise has consistently been observed to result in improvements in muscle size and function (Loenneke et al., 2012b). However, there is also evidence suggesting that BFR in the absence of exercise results in favorable muscular adaptations (Takarada et al., 2000a; Kubota et al., 2008; Kubota et al., 2011). Despite favorable effects of BFR in the absence of exercise, minimal mechanistic research exists on the BFR stimulus itself. In contrast, several mechanistic studies have been completed with BFR in combination with resistance training. To illustrate, previous research indicates that BFR combined with resistance exercise stimulates muscle protein synthesis (MPS; Fujita et al., 2007; Fry et al., 2010; Gundermann et al., 2012), although little is known about the exact cellular mechanisms associated with these changes in protein balance. It has been suggested that low intensity resistance exercise combined with BFR produces a metabolic “overload” (i.e., depletion of phosphocreatine stores and decreases in muscle pH) normally associated with higher muscle activations observed during high intensity resistance exercise (Takarada et al., 2000b; Suga et al., 2010). In addition, it has been recently observed that the benefits of BFR resistance exercise may, in part, be related to the concomitant decrease in the mRNA gene expression of MURF-1, atrogin, and myostatin (Manini et al., 2011; Laurentino et al., 2012).

Three separate investigations have noted positive results from the application of BFR in the absence of exercise. For example, Takarada et al. (2000a) observed that applying BFR (238 mmHg, 9 cm wide cuff) to patients following anterior cruciate ligament reconstruction surgery effectively diminished the post operation disuse atrophy (measured by MRI) of the knee extensors. In support of this attenuating effect, Kubota et al. (2008) found that applying BFR (200 mmHg, 7.7 cm wide cuff) to a cast immobilized limb not only attenuates decreases in muscle size (measured by leg circumference) but also muscle strength. However, a separate study from the same group indicated that a lower pressure of 50 mmHg reduced muscular weakness induced by chronic unloading, but had no effect on attenuating changes in leg size (Kubota et al., 2011). Interestingly, despite the published evidence, there has been no speculation on what mechanism(s) may be at work during BFR in the absence of an exercise stimulus. Instead, authors have applied the findings from mechanistic work completed on resistance exercise studies to explain results of the aforementioned BFR only studies.

Although reductionism plays an important and useful role in science, there are numerous problems with using this approach to mechanistically understand the effects of BFR in the absence of exercise. For example, the increases in electromyography (EMG) activity observed with low intensity resistance exercise combined with BFR are greater than those observed with resistance exercise alone (Takarada et al., 2000b; Yasuda et al., 2009). One investigation found that the EMG activity was 40% lower when exercise was completed without BFR (Takarada et al., 2000b). Using the aforementioned reasoning would lead one to suggest that part of the benefits observed with BFR in the absence of exercise are due to this increase in EMG activity, however, in the absence of muscle contraction, there are no changes in EMG activity. Furthermore, our unpublished data also suggests that no measurable changes occur in whole blood lactate, indicating that large changes in hydrogen ion concentration are likely not occurring with BFR in the absence of exercise. The only two variables investigated that did change with BFR only were acute increases in real-time ultrasound measured muscle thickness and a decrease in hematocrit determined plasma volume. These changes were maintained following the removal of the cuff suggesting that the acute changes in muscle thickness were actual acute increases in muscle size (i.e., muscle swelling) and were not attributed to venous pooling. This acute change in size has also been observed following low intensity BFR resistance training. To illustrate, Fry et al. (2010) observed greater acute increases in muscle size (measured by circumference) with BFR resistance exercise compared to resistance exercise without BFR. Interestingly, they too suggest that this might mechanistically explain part of the increase in MPS they observed following BFR resistance exercise.

Many hypotheses exist for the mechanism behind the beneficial effects observed with BFR; however it may be necessary to research the effects of BFR in the absence of resistance exercise in order to allow for a clearer picture of what is physiologically occurring with the BFR stimulus itself. We would like to suggest that acute muscle swelling may be the mechanism behind the reductions in muscle size and strength declines observed following surgery or cast immobilization. In addition, this swelling may also help explain the increases in muscle size and strength previously observed from slow walking in combination with BFR (Abe et al., 2006; Ozaki et al., 2011). Although we have non-invasive data suggesting this is true, we are left to speculate as to how the acute increase in muscle size happens and how this acute increase in muscle size might lead to favorable changes in protein balance. Previous findings suggest that cellular dehydration may be involved in the down regulation of mammalian target of rapamycin (mTOR) signaling (Schliess et al., 2006). Therefore, if BFR can acutely increase the influx of water into the muscle cell, this may provide a stimulus capable of stimulating the mTOR pathway. It is acknowledged that this swelling hypothesis is largely dependent on research completed on hepatocytes (Loenneke et al., 2012a); therefore it is unknown how well this mechanism may translate over to human skeletal muscle. Future cellular research should attempt to determine whether or not muscle swelling plays a significant role in the muscle hypertrophic signaling response in humans, which would have important clinical implications for populations contraindicated to exercise.
